# Supplementary material for: Status of countrywide laboratory services quality and capacity in primary healthcare facilities in Tanzania: Findings from Star Rating Assessment
Source: PLOS Glob Public Health. 2023 Oct 18;3(10):e0001489. doi: 10.1371/journal.pgph.0001489 (PMC10584114; doi:10.1371/journal.pgph.0001489)
Supplement: S1 Table — (DOCX) [file pgph.0001489.s001.docx]

S1 Table: Univariate analysis of the Facility’s characteristics that define the scores for laboratory quality services at Primary Healthcare, 2017-2018

| **Independent variable** | **B (Slope)** | **Standard error (SE)** | **t-ratio(t)** | **prob.(p)** | **R2** |
| --- | --- | --- | --- | --- | --- |
| Dispensary level | -0.433 | 0.010 | -41.87 | <0.001 | 0.179 |
| Rural-based facilities | -0.337 | 0.010 | -33.15 | <0.001 | 0.158 |
| Public facilities | -0.366 | 0.010 | -35.36 | <0.001 | 0.153 |
| Complying with lab staffing level | 0.512 | 0.076 | 67.43 | <0.001 | 0.402 |

R^2^ = coefficient of determination (adjusted),
