# Supplementary material for: Status of countrywide laboratory services quality and capacity in primary healthcare facilities in Tanzania: Findings from Star Rating Assessment
Source: PLOS Glob Public Health. 2023 Oct 18;3(10):e0001489. doi: 10.1371/journal.pgph.0001489 (PMC10584114; doi:10.1371/journal.pgph.0001489)
Supplement: Attachment — Submitted filename: Responses to reviewers.docx [file pgph.0001489.s002.docx]

**Reviewers comments**

PGPH-D-22-02022
Contribution of quality improvement initiative in strengthening laboratory services in Primary Healthcare Facilities in Tanzania: findings from 2017/2018 Star Rating Assessment
PLOS Global Public Health

Dear Editor, Thank you for this opportunity. After serious consideration and review; we thought the most appropriate title of this study is **Status of countrywide laboratory services quality and capacity in Primary Healthcare Facilities in Tanzania: findings from Star Rating Assessment**

Dear Dr. Kinyenje,

Thank you for submitting your manuscript to PLOS Global Public Health. After careful consideration, we feel that it has merit but does not fully meet PLOS Global Public Health’s publication criteria as it currently stands. Therefore, we invite you to submit a revised version of the manuscript that addresses the points raised during the review process.

 Thank you for consideration

Please see the comments below and in the attachment from three reviewers. Please pay special attention to their comments on the statistical analysis. Two reviewers have requested more context on the situation in Tanzania. Please note that although one reviewer suggests shortening the introduction, there are no specific word limits for PLOS Global Public Health.

Please submit your revised manuscript by Apr 30 2023 11:59PM. If you will need more time than this to complete your revisions, please reply to this message or contact the journal office at globalpubhealth@plos.org. When you're ready to submit your revision, log on to https://www.editorialmanager.com/pgph/ and select the 'Submissions Needing Revision' folder to locate your manuscript file.

Please include the following items when submitting your revised manuscript:

- A rebuttal letter that responds to each point raised by the editor and reviewer(s). You should upload this letter as a separate file labeled 'Response to Reviewers'.
- A marked-up copy of your manuscript that highlights changes made to the original version. You should upload this as a separate file labeled 'Revised Manuscript with Track Changes'.
- An unmarked version of your revised paper without tracked changes. You should upload this as a separate file labeled 'Manuscript'.

Guidelines for resubmitting your figure files are available below the reviewer comments at the end of this letter.

We look forward to receiving your revised manuscript.

Kind regards,

Hanna Landenmark
Staff Editor
PLOS Global Public Health

Journal Requirements:

Additional Editor Comments (if provided):

Reviewers' comments:

Reviewer's Responses to Questions

**Comments to the Author**

1. Does this manuscript meet PLOS Global Public Health’s [publication criteria](https://journals.plos.org/globalpublichealth/s/criteria-for-publication)? Is the manuscript technically sound, and do the data support the conclusions? The manuscript must describe methodologically and ethically rigorous research with conclusions that are appropriately drawn based on the data presented.

Reviewer #1: Yes

Reviewer #2: Yes

Reviewer #3: No

2. Has the statistical analysis been performed appropriately and rigorously?

Reviewer #1: No

Reviewer #2: No

Reviewer #3: Yes

3. Have the authors made all data underlying the findings in their manuscript fully available (please refer to the Data Availability Statement at the start of the manuscript PDF file)?

The [PLOS Data policy](https://journals.plos.org/globalpublichealth/s/data-availability) requires authors to make all data underlying the findings described in their manuscript fully available without restriction, with rare exception. The data should be provided as part of the manuscript or its supporting information, or deposited to a public repository. For example, in addition to summary statistics, the data points behind means, medians and variance measures should be available. If there are restrictions on publicly sharing data—e.g. participant privacy or use of data from a third party—those must be specified.

Reviewer #1: No

Reviewer #2: No

Reviewer #3: Yes

4. Is the manuscript presented in an intelligible fashion and written in standard English?

PLOS Global Public Health does not copyedit accepted manuscripts, so the language in submitted articles must be clear, correct, and unambiguous. Any typographical or grammatical errors should be corrected at revision, so please note any specific errors here.

Reviewer #1: Yes

Reviewer #2: Yes

Reviewer #3: No

5. Review Comments to the Author

Please use the space provided to explain your answers to the questions above. You may also include additional comments for the author, including concerns about dual publication, research ethics, or publication ethics. (Please upload your review as an attachment if it exceeds 20,000 characters)

Reviewer #1: The paper presents an important perspective on Quality control of laboratories in PHC settings. However, it needs to address some key issues.

Abstract: Methods should mention the key domains of assessment and score. Results should mention % of \dispensary/rural/ and private (given in the main body). Association of total lab score with these factors should be mentioned.

Thank you for this observation. Indicators used to determine the quality scores for laboratory services have been provided. In summary, we have commented on how data were analysed and categorized. Proportions of the facilities based on facility level (dispensaries, health centres, hospital level1), ownership (public, private), location (urban, rural) has been added in the abstract.

Introduction:

it is too long and needs to be edited to half its length. First two paragraph can be deleted/shortened and full reference to a WHO report is not needed in the main text.

Thank you for this observation. The reference to the WHO report and framework has been rephrased. Also, for noting is that Tanzania from 2007 to 2017 embarked on a very extensive programme to strengthen primary health care (PHC) services called “primary Health Services Development Programme”; and other interventions were added as learning through the reforms process & health system strengthening emerged. Therefore, the length of the Introduction part (especially the two paragraphs) is essential to ensure that we are cognizant of what targeted interventions have been there so that the findings of our study (paper) can enable decision-makers to narrow and focus on the specific gaps that we have revealed. This will help PHC in Tanzania to move to a further step in particular as the country zero on concerted efforts towards achievement of universal health coverage (UHC).

Methods: Current table 1 has to be reorganized. First column can be deleted. And the description of each domain currently in the text can be shifted here as an additional column.

Thank you for the advice. The first column is deleted and the description of each indicator is now in additional column.

One main problem with the paper is the regression. Since each of the domain score contributes to the total score, they cannot be taken as independent variable. it is no surprise that 99.1% of the variance is explained. It is also apparent from the fact that the domains with more items correlate better to the total score. It is inappropriate to do this. They can keep variables like rural/urban, dispensary/others, private/public etc. in the regression equation.

Thank you for this important observation. We surely overlooked the condition for independence among variables contributing to dependent variables. Variables location (rural/urban), facility level (dispensary/health centre/hospital), ownership (private/public) AND laboratory staffing level are now considered as new independent variables. They are all termed as facility characteristics.

The total score and domain specific score can be shown (table 4 - first column can be deleted) . Since staff are important inputs one separate table (table 2 & 3 can be combined) can be provided.
The then Table 2 described more about how data were collected to get into “laboratory staff compliance”. We think this is a repetition of the statements in the methodology section (Refer to Table 1). We believe a detailed Table 2 (which was then Table 3) will provide adequate information regarding staff compliance.

Discussion may be rewritten accordingly.

This comment is well received. We have improved our discussion part to include all of your suggestions.

Reviewer #2: 1. Describe the study area. i.e., Tanzania.
1.1. For the global audience to connect with the research, it is important to describe the Tanzania as a nation. This description could be used to put it in the global perspective. For example, if economic indicators like GDP, per capita income are used then it could be used to define if the country is a Low Income country, Middle Income Country or High Income country based on global standards. Some indexes that should be provided are HDI, SDG ranking, Population demographics, social indicators, literacy rate, per capita income, GDP, etc. It could be provided as separate section or sub-section of Methods.

Thank you for this important comment. A sub-section in methods section describing all of the above has been added.

1.2. In the discussion section, compare the laboratory status of Tanzania with other similar nations from literature to better understand the gravity of situation in global context. For example, compare the staffing level status in Tanzania with staffing level in other similar nations. Please provide reasons why it performs better, similar, or worse than other nations.

Thank you for this important contribution. We have improved our discussion part to include all of your suggestions.

2. Explain the dependent variable, i.e., SRA.
2.1. In the methods section, please explain the SRA in more detail:
2.1.1. How is it calculated? Do they sum the values from all the 12 criteria? How are the laboratory scores added?
2.1.2. What is the outcome type? Is it a Likert scale, categorical scale or continuous outcome?

Thank you for this important comment. Description on how dependent variables has been added in “Management of Study variables and Statistical analysis” sub-section. However, the description has been preceded by detail description on how the data that make up the variables were collected and managed

2.2. In the results section, please provide the distribution profile of SRA. For example, if it is continuous outcome, please provide mean, median, mode, range, standard deviation, etc. If categorical/Likert, please provide number of datapoints (or, percentage of datapoints) under each category? Is it a uniform distribution for categorical outcome?

Thank you for the reminder. The distribution profile of the dependent variable has been given.

3. Please explain why Chi square test is not performed for section “The proportion of PHCs that comply with indicators that measure the quality of laboratory services”?
3.1. Please perform the Chi square test for the section to maintain the consistency in the paper.

Thank you for the comment. We performed the chi-square test and found all pairs had p-value of less than 0.001. Therefore, to avoid Table 4 being busy, we wrote the following general note below the table: “*A chi-square test was performed to each indicator-facility level pair and p-value of <0.001 was found across all pairs.”*

4. In Table 4, please provide the overall percentage for following sections:
4.1. 12.2.4
4.2. 12.2.6
4.3. 12.2.7

Thank you for the comment. However, the above items were the broad themes from which we have specific variables. Summing up the specific variables within the broad theme equals not to the score of the broad theme. So to avoid confusion we preferred to rely on specific variables. Table 4 is now Table 3

5. Please provide the facility level distribution of outcomes in Table 4. This could be provided in supplementary section.

Your suggestion is good. We have modified columns in Table 4 (currently Table 3) to provide the facility-level distribution of the outcome.

6. In the regression model, the r square value of 0.99 needs more clarification.
6.1. Does it indicate that other parameters, i.e., non-laboratory parameters used in preparing the STAR rating do not influence the STAR rating?

Thank you for this important observation. We surely overlooked the condition for independence among variables contributing to dependent variables. Variables location (rural/urban), facility level (dispensary/health centre/hospital), ownership (private/public) AND laboratory staffing level are now considered as new independent variables. They are all termed as facility characteristics. The current r value is 57.1%. this means non laboratory influence the score by more than 40%

6.2. Please perform the univariate analysis with all the independent variables.

Thank you for this observation. The univariate analysis with the independent variables has been performed and attached as “S1 Appendix”

6.3. Please prepare the control model with only facility characteristics.

Thank you for this important comment. We thought after working on issues related to variables and excluded the rest of the variables except one plus facility characteristics then we may not need the control model. We did a control model to see whether it gives different association. There was no change in association but the magnitude. If you think the model is still necessary, please we will take that seriously.

Reviewer #3: The manuscript was presented in such a way that makes it challenging to mention the portion i was talking about. because it would have been nice if all lines were numbered. Some operational definition or explanation in the back ground has to be included to clearly show some one who don't know the system in Tanzania. Other wise the statistical analysis looks good except few comments given in attachment.

Thank you for the comment. We have included more information about Tanzania in manuscript e.g. Study area section

6. PLOS authors have the option to publish the peer review history of their article ([what does this mean?](https://journals.plos.org/globalpublichealth/s/editorial-and-peer-review-process#loc-peer-review-history)). If published, this will include your full peer review and any attached files.

**Do you want your identity to be public for this peer review?** If you choose “no”, your identity will remain anonymous but your review may still be made public.

For information about this choice, including consent withdrawal, please see our [Privacy Policy](https://www.plos.org/privacy-policy).

Reviewer #1: **Yes:** Anand Krishnan

Reviewer #2: No

Reviewer #3: No
