# Supplementary material for: Status of countrywide laboratory services quality and capacity in primary healthcare facilities in Tanzania: Findings from Star Rating Assessment
Source: PLOS Glob Public Health. 2023 Oct 18;3(10):e0001489. doi: 10.1371/journal.pgph.0001489 (PMC10584114; doi:10.1371/journal.pgph.0001489)
Supplement: Attachment — Submitted filename: responses to reviewers.docx [file pgph.0001489.s003.docx]

**Reviewers comments**

PGPH-D-22-02022
Contribution of quality improvement initiative in strengthening laboratory services in Primary Healthcare Facilities in Tanzania: findings from 2017/2018 Star Rating Assessment
PLOS Global Public Health

PGPH-D-22-02022R1
Status of countrywide laboratory services quality and capacity in Primary Healthcare Facilities in Tanzania: findings from Star Rating Assessment
PLOS Global Public Health

Dear Dr. Kinyenje,

Thank you for submitting your manuscript to PLOS Global Public Health. After careful consideration, we feel that it has merit but does not fully meet PLOS Global Public Health’s publication criteria as it currently stands. Therefore, we invite you to submit a revised version of the manuscript that addresses the points raised during the review process.

The three previous reviewers were available to comment on your revised manuscript. Two of the reviewers are more positive about this new version, but the other reviewer has raised remaining concerns about your manuscript that need to be addressed. Pay attention to the comment that there have been three previous national surveys on this subject, and contextualise your study in the context of this previous research.

Please submit your revised manuscript by Jul 07 2023 11:59PM. If you will need more time than this to complete your revisions, please reply to this message or contact the journal office at globalpubhealth@plos.org. When you're ready to submit your revision, log on to https://www.editorialmanager.com/pgph/ and select the 'Submissions Needing Revision' folder to locate your manuscript file.

Please include the following items when submitting your revised manuscript:

- A rebuttal letter that responds to each point raised by the editor and reviewer(s). You should upload this letter as a separate file labeled 'Response to Reviewers'.
- A marked-up copy of your manuscript that highlights changes made to the original version. You should upload this as a separate file labeled 'Revised Manuscript with Track Changes'.
- An unmarked version of your revised paper without tracked changes. You should upload this as a separate file labeled 'Manuscript'.

Guidelines for resubmitting your figure files are available below the reviewer comments at the end of this letter.

We look forward to receiving your revised manuscript.

Kind regards,

Miquel Vall-llosera Camps
Staff Editor
PLOS Global Public Health

Journal Requirements:

1. Please send a completed 'Competing Interests' statement, including any COIs declared by your co-authors. If you have no competing interests to declare, please state "The authors have declared that no competing interests exist". Otherwise please declare all competing interests beginning with the statement "I have read the journal's policy and the authors of this manuscript have the following competing interests:"

[Note: HTML markup is below. Please do not edit.]

Reviewers' comments:

Reviewer's Responses to Questions

**Comments to the Author**

1. If the authors have adequately addressed your comments raised in a previous round of review and you feel that this manuscript is now acceptable for publication, you may indicate that here to bypass the “Comments to the Author” section, enter your conflict of interest statement in the “Confidential to Editor” section, and submit your "Accept" recommendation.

Reviewer #1: (No Response)

Reviewer #2: All comments have been addressed

Reviewer #3: All comments have been addressed

2. Does this manuscript meet PLOS Global Public Health’s [publication criteria](https://journals.plos.org/globalpublichealth/s/criteria-for-publication)? Is the manuscript technically sound, and do the data support the conclusions? The manuscript must describe methodologically and ethically rigorous research with conclusions that are appropriately drawn based on the data presented.

Reviewer #1: Partly

Reviewer #2: Yes

Reviewer #3: No

3. Has the statistical analysis been performed appropriately and rigorously?

Reviewer #1: Yes

Reviewer #2: Yes

Reviewer #3: Yes

4. Have the authors made all data underlying the findings in their manuscript fully available (please refer to the Data Availability Statement at the start of the manuscript PDF file)?

The [PLOS Data policy](https://journals.plos.org/globalpublichealth/s/data-availability) requires authors to make all data underlying the findings described in their manuscript fully available without restriction, with rare exception. The data should be provided as part of the manuscript or its supporting information, or deposited to a public repository. For example, in addition to summary statistics, the data points behind means, medians and variance measures should be available. If there are restrictions on publicly sharing data—e.g. participant privacy or use of data from a third party—those must be specified.

Reviewer #1: Yes

Reviewer #2: No

Reviewer #3: Yes

5. Is the manuscript presented in an intelligible fashion and written in standard English?

PLOS Global Public Health does not copyedit accepted manuscripts, so the language in submitted articles must be clear, correct, and unambiguous. Any typographical or grammatical errors should be corrected at revision, so please note any specific errors here.

Reviewer #1: Yes

Reviewer #2: Yes

Reviewer #3: Yes

6. Review Comments to the Author

Please use the space provided to explain your answers to the questions above. You may also include additional comments for the author, including concerns about dual publication, research ethics, or publication ethics. (Please upload your review as an attachment if it exceeds 20,000 characters)

Reviewer #1: Introduction is too long and needs to be condensed to half. Unnecessary details in methods section on SDGs/HDI is not required.
Thank you for the comment. Many details in the method section on SDGs/HDI were once requested by an earlier reviewer. However, we have rephrased some of the sentences to remove some unnecessary information.

The paper uses data from a report, probably in public domain, and the value of addition of this paper is not clear. It is also understood that there have been three such national surveys with the most recent being 2020-21. I think the value addition would be to see the change in the laboratory services in Tanzania over time and link it to the interventions. Without that this paper publication value is low.

Thank you for this comment, which gives us an opportunity to provide a clear statement on the value addition of this paper. This paper presents the first-ever nationwide assessment of laboratory services at primary healthcare facilities. The assessment is typically extensive and time-consuming, taking about two years to complete. Although the data used in this study may appear outdated (2017/18), considering the scale of the exercise relative to the country's capacity, these statistics are highly beneficial in informing policy and decision-making. The assessments that we initiated in 2021/2022 are still ongoing nationwide, and we will utilize them later, upon completion, to compare the progress of services in the country.

In most places, scores are ‘0’ and ‘1’. Could the authors consider them as nothing – 0, partial – 1 and complete – 2 to give better sensitivity to the score.

Among the seven indicators of laboratory services included in this study, only three indicators were measured using three values: Yes, partial, and No. Other indicators had values of either Yes or No. This was due to the design of the assessment tool, which involved international experts. However, we consider this as a challenge and an opportunity to improve our tool for future assessments, making it more sensitive.

Table 1 is not required.

Thank you for this important suggestion. However, along with this excellent suggestion, we thought that using a table instead of free text would help improve the organization and flow of concepts. We also believed that the table has reduced the wordiness yet remained comprehensive, enhancing readability as well. If this matter is still unsatisfactory, we are willing to improve. Thank you.

The outcome variable for the linear regression was the crude score or the percentage score out of maximum. It should be noted that dispensary had a lesser maximum score than the health centers and hospitals.

The variable used as the outcome in linear regression analysis was the percentage obtained by dividing the crude score by the total or maximum score. This was significant in order to establish a relative comparison among various levels of healthcare facilities.

Reviewer #2: Almost all the modifications have been done in the article. Thus, it can be accepted. However, I would like to provide mere suggestions based on more clarity provided in the manuscript. No additional review is needed if authors choose to work on these suggestions.
• Author is encouraged to provide the control model, it could be provided as supplementary file, which will help the readers to get the complete picture of the variables

Thank you so much for your positive comments on our work. In our opinion, we believed that linear regression analysis was a sufficient statistical technique to control for the influence of specific variables. We included various variables as covariates in the analysis, which allowed us to assess the independent effect of the variable of interest on the outcome. However, we are open to guidance if you still think there are further improvements needed.

• Authors research is addressing a key issue in healthcare system. Laboratories need the focus in the healthcare system and policies should be more proactive. However, the idea “diagnostic services are an essential ingredient of the UHC target” could be strengthened with some references. One of the key ideas to strengthen this argument is to provide some quantitative relationship between laboratory and policy goals (sub-national, national or international). For example, in the following papers authors have tried to establish a quantitative relationship between laboratories and overall facility/policy performance.
o https://www.emerald.com/insight/content/doi/10.1108/09564230010340715/full/html
o https://www.ncbi.nlm.nih.gov/pmc/articles/PMC6635801/
Authors are encouraged to refer these or similar research papers in their policy recommendation section to provide a stronger justification for the need to focus on the issues identified by the authors.

Thank you for this comment. We have referred the papers you provided and one more to enrich the section on policy recommendation. One more recommendation has been added.

Reviewer #3: The authors have gone through each comments well and revised what they need to. Now what may be need to added is just they need to re arrange it to the publication format which of course the editors will go through. other wise i am comfortable with the paper.

Thank you so much, we appreciate it.
