# Supplementary material for: Status of countrywide laboratory services quality and capacity in primary healthcare facilities in Tanzania: Findings from Star Rating Assessment
Source: PLOS Glob Public Health. 2023 Oct 18;3(10):e0001489. doi: 10.1371/journal.pgph.0001489 (PMC10584114; doi:10.1371/journal.pgph.0001489)
Supplement: Attachment — Submitted filename: 2nd review (1).docx [file pgph.0001489.s004.docx]

Contribution of quality improvement initiative in strengthening laboratory services in Primary Healthcare Facilities in Tanzania:

Manuscript Number: PGPH-D-22-02022

Corresponding Author: Erick S. Kinyenje et al.

1. The manuscript was difficult to comment as the lines were not numbered just as the the older version.
2. Again as just last version comment which is not corrected by the authors is study population should tell us. which health facilities are included, from which health facilities data extraction taken place? How the selection of PHC were taken place, what method was used to identify this health facilities from whole? if the authors took the whole health facilities in the country it okay and mention it otherwise still the explanation it needs correction.
3. Does qualified laboratory have operational definition? What is the criteria to say qualified? Operationalize it on this paper please.to label qualified which or how many indicators are expected to fulfil?
